# Introduction

**DOI:** 10.1080/11287462.2021.2011000

**Published:** 2022-02-06

**Authors:** Cristina Richie

**Affiliations:** Ethics and Philosophy of Technology Section, TU Delft

Every day from the moment we wake up until the moment we sleep, we have the option to look outward or look inward. This is more than an outlook; it is also a metaphor. As we – citizens, ethicists, clinicians, health professionals, lawyers, theologians, academicians, researchers, and educators – prepare for our day we choose our disposition to our world, in fact, our worldview.

Those who look outward are mindful of their surroundings, their responsibilities, their obligations, and indeed their place in the world, from the social microcosm of their city to their contribution to their nation, to their impact on the ecosystem. Concretely, this affects their engagement in fundamental aspects of social life – commerce and recreation; privacy and technology; transportation and energy; participation in just institutions, and active reform of racist, sexist, and heterosexist structures.

Those who look inward are also in a microcosm, often facilitated by the immediate and persistent attachment to handheld digital devices. The din of self-imposed boundaries of exclusivity between the agent and other dignified beings restricts intellectual movement, creating a recursive echo chamber that is difficult to escape, as it is self-contained and self-created.

The metaphor of outward and inward attitudes extends to collective bodies, like committees, task forces, groups of affinity, and executive boards. It extends to concepts like “bioethics” and “globalization” as well.

Conceptually, bioethics – constructed by Fritz Jahr and Sass (2010) and later Van Rensselaer Potter (1982, 1988) – was outward looking. It addressed the “problems of interference with other living beings … and generally everything related to the balance of the ecosystem” (Reich, 1978, introduction, xix). The “bilocated birth” of bioethics/biomedical ethics need not be rehashed here (Reich, 1995), though it is worth noting that the discipline continues to fracture and suffer from identity politics. With the recent co-optation of “biomedical ethics” by clinical ethics – the latter of which has a significant interest in the monetization of ethics – the discipline as a whole is spiraling closer to an *égoïsme à deux* between patient and the medical industrial complex instead of maintaining an expansive, even politically aware stance as an ethical system in service to the common good. Particularly as even those who are outside the traditional medical setting are scanned as potential patients, subjected to the medical gaze as businesses decide who is a health risk based on their ability to procure a *pass sanitaire* (French: health pass), the integrity of bioethics not only as liberation from medical imposition (hence the principle of respect for autonomy) but also an ethical philosophy dedicated to solidarity and justice is further compromised.

Thus, we seek here a return to the concept of bioethics, which requires reaffirmation of its global nature. Bioethics was always “global,” holistic, multifaceted. Global does not alter – but rather affirms – Jahr and Potter’s vision. In this way, it ideologically conserves, but not preserves, as bioethics is not a petrified forest or museum of dead thoughts. Hence, our vision for global bioethics is outward, forward, upward. It is nascently contained here in the selection of essays for this, our inaugural issue, under the new editorial leadership of Cheryl Cox Macpherson and Cristina Richie.

Ruth Macklin’s lead article “A New Definition for Global Bioethics: COVID-19, a Case Study” describes the “relations between and among countries … (as) a clue to what is needed for understanding what global bioethics is and does.” In particular, her case study on Covid-19 and the insulated response of many countries to the Covid pandemic in the form of “vaccine nationalism” highlights domestic inwardness. Macklin writes that vaccine equity agreements, which “began as a good-faith effort to allocate vaccines in an equitable manner quickly gave way to self-interested behavior on the part of rich and powerful nations.”

Self-interested behavior, of course, is not limited to pandemic responses but is symptomatic of inward-looking countries. Yet, as Gustavo Ortiz Millán’s commentary on Macklin’s article suggests, “a more globalized world presents bioethics with new challenges; cases that call for a global response and also a global bioethics.” These challenges include wildlife poaching and trade, zoonotic outbreaks, biodiversity loss, and climate change. His call in “Bioethics, Globalization and Pandemics” that “global bioethics should take the concept of One Health more seriously” extends to poverty-based global health disparities as well as integrative approaches to health for people, animals, and the environment.

Rosemary Tong’s “Towards a Feminist Global Ethics” outlines the impacts of globalization on both feminism and women by carving out “room in global bioethics for a certain type of non-imperialistic, non-colonial universalism that is present in some formulations of *feminist* global bioethics” (italics hers). She, too, observes the splits in feminist thought between values like autonomy and community, which tend to fall along Western/non-Western lines, respectively. The often inward-looking agenda of Western feminism is at odds with the outward-facing decolonization of both feminism and ethics, and thus both humanism and bioethics. Therefore, instead of balking at concepts, such as care, gender essentialism, and interdependence, a global bioethics is truly inclusive since it values these ideas in addition to justice, gender constructivism, and independence. To be sure, the dichotomy of outward/ inward does not fall predictably on other dyads. Eva Feder Kittay’s characterization of “rights/justice-based ethics/bioethics on the one hand and relationships/care-based ethics/bioethics on the other hand,” noted in Tong’s essay, could be both inward or outward in application.

Indeed it is just this pluralistic, non-binary approach that Christine Overall presents as she interrogates “The Role of Care” in her commentary on Tong’s article. Since “looking at the lengthy list provided by Dr. Tong of issues that global bioethics deals with it is hard to see how care can resolve them,” Overall suggests that “in order to develop a feminist global bioethics, what is needed is an *intersectional* approach to the values of care and justice” (italics hers). In doing so, ethical dilemmas may be solved and resolved, thus widening the aperture of bioethical impact.

In taking perhaps the broadest (re)interpretation of bioethics, Henk ten Have observes, “the phenomenon of a pandemic clearly reactivates the notion of global bioethics” by facilitating the opportunity to address issues typically overlooked by (non-global) bioethics, like “community, the common good, solidarity and fairness.” His article “The Challenges of Global Bioethics” distills both Macklin and Tong’s articles, thus poising global bioethics for significant methodological work in both scope – that is, problems of a “global nature … intimately connected to processes and practices of globalization which have characterized international relations” – and the moral orientation of “unity of humanity, solidarity, equality, openness to differences, and focus on what human beings have in common.”

Taken in aggregate, the articles which were solicited for our thematic issue, which attempts to re-define global bioethics and “deepen understandings of what ‘global bioethics’ is and does” simply does just that. Global bioethics connects rather than separates; builds paths rather than blocks them. Global bioethics replicates the intricate matrixes found in nature as both a holistic ethical system and as a spatial location. As such, our vision for the future of the journal henceforth explores the many facets of global bioethics – with an eye towards effecting significant change in the most pressing and perennial ethical issues of the *unwelt*. We welcome dialogue and contributions in this endeavor.
